# Photorealistic avatars can help to improve treatment outcome expectations

**DOI:** 10.3389/fpsyg.2026.1960478

**Published:** 2026-10-21

**Authors:** Pauline Bauermann, Winfried Rief

**Affiliations:** Department of Clinical Psychology and Psychotherapy, Marburg University, Marburg, Germany

**Keywords:** alliance, avatars, expectation violation, outcome expectations, sequence effects

## Abstract

**Introduction:**

Because negative treatment outcome expectations predict unfavorable treatment effects, violating these negative outcome expectations is one central goal of psychotherapy. One mediator of outcome expectations and therapy outcome is the therapeutic alliance.

**Methods:**

This experimental online study investigated whether human vs. photorealistic avatar therapists can establish a therapeutic alliance through a first empathy video followed by an expectation violation video, challenging negative treatment expectations. Healthy participants (*N* = 484) were assigned to one out of four experimental groups where either a human therapist or their photorealistic avatar expressed empathy (video 1) and gave expectation-violating information (video 2).

**Results:**

In line with our hypothesis (*two human therapists* > *human*, *then avatar therapist* > *avatar*, *then human therapist* > *two avatar therapists)*, outcome expectation improvements and alliance ratings were highest after the two videos for the sequence with two human therapists, and still considerably positive in the groups with the avatar in the first video.

**Discussion:**

Contrary to our hypothesis, outcome expectations and alliance ratings declined after the second video for the group that first saw a human and then an avatar therapist: rather than potentiating the expectation violation after a *human* alliance, a negative contrast effect could have occurred when seeing an avatar after an initial human therapist, besides a modest, general effect of avatar-delivered content. When combining human and avatar therapist modalities in a single session, it seems important to consider the sequence of avatar and human therapist display: targeted expectation violation effects may be stronger when the human therapist follows the avatar.

## Introduction

While the efficacy of traditional psychotherapy is well-established (e.g., Harrer et al., 2025), app-supported mental health interventions have also proven effective for a variety of conditions, offering a cost-efficient alternative to face-to-face therapy (Linardon et al., 2019; Kählke et al., 2022; Andersson et al., 2014; Ebert et al., 2019). Negative outcome expectations can lead to premature termination and reduced adherence to therapy (Nasiri-Dehsorkhi et al., 2024), and predict lower treatment success (*r* = 0.18; Constantino et al., 2018; see also Rozenkrantz et al., 2025). This shows that it can be fruitful to monitor outcome expectation trajectories and foster positive outcome expectations from the outset, as well as respond sensitively when outcome expectations decline (Constantino et al., 2018). Outcome expectations predicted clinical outcomes to the same degree in online and face-to-face interventions (Pontén et al., 2024). Violating negative outcome expectations can be seen as one crucial goal of psychotherapy (Rief et al., 2022). A cost-effective approach may involve violating negative outcome expectations through photorealistic avatars when beginning psychological treatment.

Therapeutic alliance is a partial mediator between outcome expectations and therapy outcome, such that more positive outcome expectations were associated with higher alliance, which in turn was associated with better therapy outcomes (Constantino et al., 2020). Meta-analyses show a robust association between alliance and therapy outcomes (*r* = 0.278), with a comparable effect size for Internet-based psychotherapy (*r* = 0.275) (Flückiger et al., 2018) and blended care formats (Askjer and Mathiasen, 2021). Grodniewicz and Hohol (2023) argue that, despite the absence of a human therapeutic relationship, non-human agents may deliver therapeutic interventions more consistently than humans, and that “active” components of human-human relationships such as empathy, trust, and positive regard can be reconstructed within human-agent interaction (Grodniewicz and Hohol, 2023). In a meta-analysis, face-to-face interventions showed stronger results for the *goal* dimension of the therapeutic alliance, whereas blended interventions showed stronger results for the *task* dimension (Askjer and Mathiasen, 2021). The question arises as to how blended care interventions should be structured to ensure an optimal therapeutic alliance and subsequent violation of negative outcome expectations.

Blended treatments can be defined as “treatment programs that use elements of both face-to-face and Internet-based interventions” (Erbe et al., 2017, p. 2). Blended care therapy led to higher overall acceptance (91%), in contrast to digital therapy alone (Ferrao Nunes-Zlotkowski et al., 2024). Moreover, blended therapy for depression was noninferior or more effective compared to treatment as usual with moderate to large effect sizes (Cohen’s *d* = 1.1) (Ferrao Nunes-Zlotkowski et al., 2024).

In *sequential* designs of blended care, the Internet-based part is positioned either before or after the face-to-face treatment, as stepped care (Internet-first) or as aftercare (human-therapist-first; Erbe et al., 2017). This sequencing is central to the question of whether digital- or human-led components should come first. Early evidence suggests that the order matters, but no distinct meta-analytical classification is possible yet: Among the five studies that provided internet-based interventions while participants waited for face-to-face treatment, two found better outcomes in the intervention group. The same ratio, with two out of five studies, was found for lower relapse rates after internet-based aftercare compared to treatment as usual (Erbe et al., 2017). In summary, internet-delivered components can be effective both before and after human-led treatment, but there are currently no direct comparisons for outcome expectations and alliance. Based on the “original” therapeutic alliance (Bordin, 1979), we assume that an initial human alliance has a more positive effect on a subsequent breach of expectations than an initial alliance with a digital tool. The question then becomes: which digital medium is best suited for this role, and why avatars in particular?

Avatars are appealing surrogates because, due to their human-like appearance, they potentially send more signals relevant to building alliances than text- or chatbot-based tools. Previous studies have shown that a computer-animated avatar elicited greater enjoyment than a video recording of a real person (Dai and MacDorman, 2018), whilst similar results were found regarding trust in a hyper-realistic avatar compared with a real person (Schiefelbein, 2023). Warmth and competence are considered the cognitive and emotional components of therapeutic alliance (Howe et al., 2019; Seewald and Rief, 2023b), and we hypothesize that photorealistic therapist avatars are more effective than cartoon avatars at conveying these components, e.g., through their human-like appearance. Findings on avatar-based interventions in healthcare are mixed: avatars sometimes were preferred over chatbots, possibly due to perceived human-likeness (anthropomorphism) (Sestino and D’Angelo, 2023; Anisha et al., 2024), while in another study, a chatbot was rated more user-friendly than an avatar (Thunström et al., 2024). So far, most avatar-based interventions have proven effective for a wide range of conditions (e.g., schizophrenia, cancer, and depression) and have demonstrated positive effects on patients’ mental health as well as user satisfaction (Franco et al., 2021). The uncanny valley theory suggests that agents that are more human-like are perceived as more likable (Mori et al., 2012). In the ‘uncanny valley’, avatars provoke a sense of aversion when they are almost, but not quite, realistic (Seymour et al., 2021). Our photorealistic avatar has been designed to make the most of the advantages of anthropomorphism while avoiding the pitfalls of the ‘uncanny valley’. The narrative review of Rehm et al. (2016) proposes several advantages of avatars in psychotherapy, such as the establishment of a virtual therapeutic alliance and reduced communication barriers. These features suggest avatars may be especially effective in early or low-threshold phases of a sequential design, for example, reducing barriers to initial engagement and reducing negative outcome expectations before a human therapist continues the process.

The research question of this study is whether photorealistic avatars can reduce negative therapy expectations and build alliance. Moreover, the optimized sequence of avatar- and human-based videos is still unclear. Specifically, our hypothesis is that a purely human intervention is most likely to result in a strong violation of negative outcome expectations, whereas a purely avatar-based intervention should result in the least violation of negative outcome expectations. For blended formats, our hypothesis is that an initial human alliance performs better when an avatar subsequently violates negative outcome expectations than the reverse scenario (*Human_Avatar* > *Avatar_Human*), because a human interaction can pave the way to a stronger treatment alliance, which amplifies targeted violations of negative outcome expectations (Seewald and Rief, 2023b).

## Methods

The study was pre-registered (10.17605/OSF.IO/B7W5A) and approved by the local ethics committee (reference number: 2024-59k).

### Participants

Participants were recruited via university mailing lists and an online platform (Prolific) from March 2025 until August 2025. According to the power analysis (R-package mlmpower; Keller, 2024), assuming a small effect size of the fixed interaction effect (time × group), and a power of 1 − ß = 0.83, a sample of 480 people was to be targeted. Of the 632 people who clicked on the link to the questionnaire, 484 were analyzed (see Figure 1 in the Supplement). The final sample included 259 women, 221 men, and 4 gender-diverse people (*M* = 34.43 years, SD = 12.02; range = 18–74 years). Inclusion criteria, applied via Prolific’s filters, were: no hearing or visual impairment, adequate language skills, and no current psychotherapy. Participants who reported psychotic disorders or severe neurological disorders were excluded at the beginning of the study. Demographic data and baseline data for the four groups can be found in Table 1.

**Figure 1:**
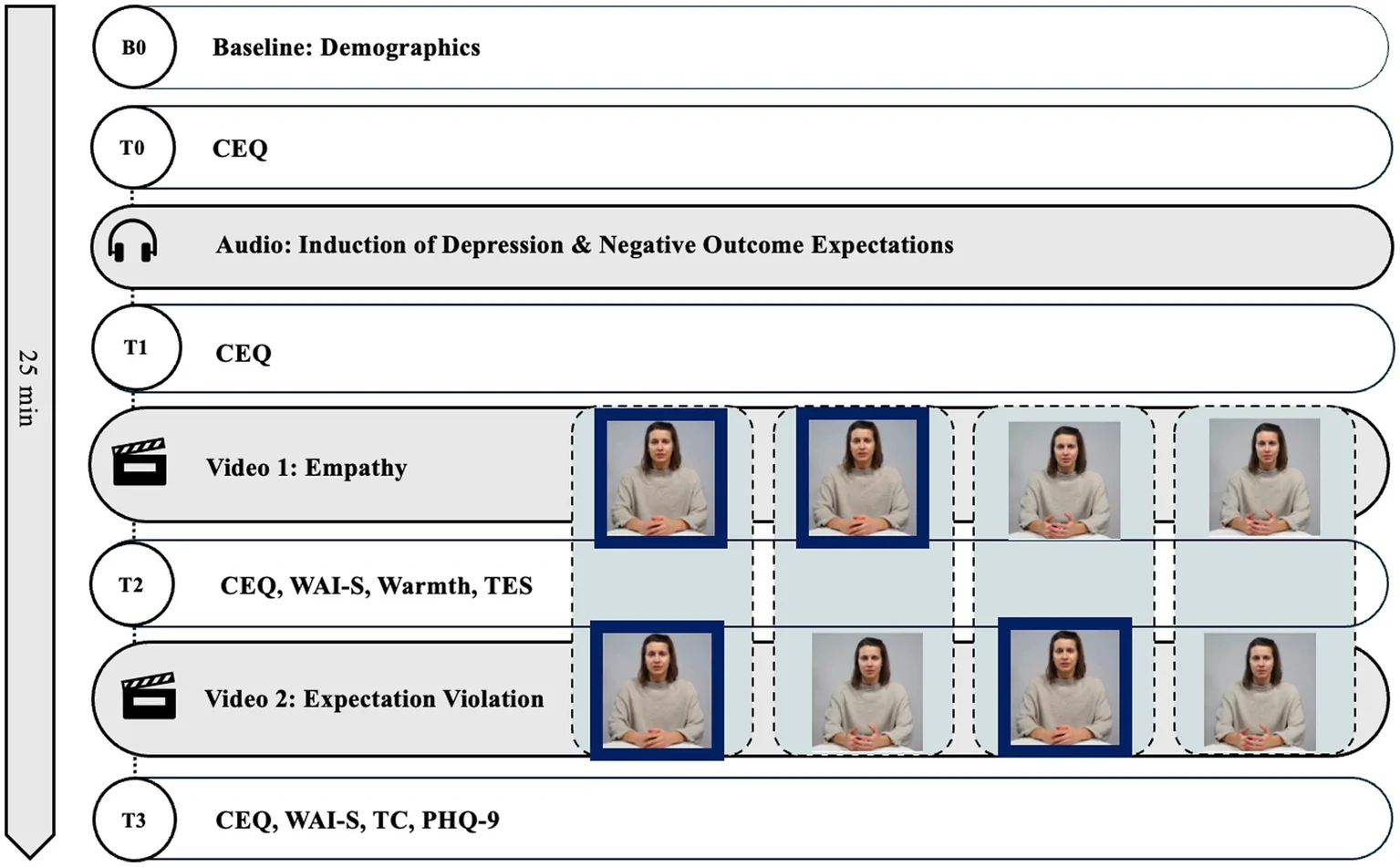
Design of the online experiment. This figure displays the experimental design for the four experimental groups *Human_Human*, *Human_Avatar*, *Avatar_Human*, and *Avatar_Avatar*. The blue box means “human.” The photorealistic avatars were less synchronized and showed more stereotypic reactions compared to the human video clips. CEQ, Credibility Expectancy Questionnaire; WAI-S, Working Alliance Questionnaire—Short; TES, Therapist Empathy Scale; PHQ-9, Patient Health Questionnaire; TC, Technology Commitment Short Scale.

**Table 1:** Sociodemographic characteristics of participants at baseline and main outcome variables at each point of time for the four groups.

Group	Human Human	Human Avatar	Avatar Human	Avatar Avatar
	*n* = 117	*n* = 120	*n* = 123	*n* = 124
Age (years) *M* (SD)	34.03 (11.91)	36.23 (13.46)	34.10 (10.79)	33.40 (11.77)
Female gender % (*N*)	52.99% (62)	53.33% (64)	54.47% (67)	53.23% (66)
College degree % (*N*)	47.01% (55)	54.17% (65)	56.10% (69)	49.19% (61)
German nationality only % (*N*)	74.36% (87)	73.33% (88)	73.17% (90)	73.39% (91)
Previous depression treatment % (*N*)	17.95% (21)	18.33% (22)	17.07% (21)	16.94% (21)
PHQ-9 *M* (SD)	6.20 (4.21)	7.23 (4.96)	7.59 (5.35)	6.78 (5.06)
Technology commitment *M* (SD*)*	3.77 (0.61)	3.83 (0.63)	3.92 (0.64)	3.92 (0.66)
Credibility fictional situation *M* (SD*)*	52.51 (29.68)	51.79 (31.51)	47.62 (32.33)	44.03 (33.35)
Previous online therapy experience % (*N*)	11.11% (13)	11.67% (14)	13.82% (17)	15.32% (19)
CEQ *M* (SD)				
T0	6.20 (1.49)	6.32 (1.24)	6.25 (1.59)	6.22 (1.63)
T1	4.89 (1.87)	5.35 (1.83)	5.20 (1.83)	5.25 (1.95)
T2	5.86 (1.76)	6.16 (1.44)	5.77 (1.77)	5.59 (2.01)
T3	6.41 (1.56)	6.03 (1.64)	6.41 (1.72)	6.19 (1.84)
WAI-S *M* (SD)				
T2	44.72 (7.47)	45.46 (7.48)	40.12 (10.76)	41.09 (9.82)
T3	45.19 (8.26)	41.52 (10.09)	42.56 (10.27)	41.52 (10.63)
Warmth *M* (SD)				
T2	3.96 (0.79)	3.27 (1.00)	3.84 (0.80)	3.53 (0.96)
Competence *M* (SD)				
T2	4.09 (0.73)	3.67 (0.91)	3.98 (0.78)	3.79 (0.93)
Empathy *M* (SD)				
T2	4.91 (1.12)	5.06 (0.99)	4.28 (1.43)	4.17 (1.38)

PHQ-9, Patient Health Questionnaire (Kroenke and Spitzer, 2002; range: 0–27); Technology Commitment (Neyer et al., 2012; scale: 1–5); Credibility of the fictional scenario (Mayer et al., 2024; range: 1–101); CEQ, Credibility Expectancy Questionnaire (Devilly and Borkovec, 2000; scale = 1–9); WAI-S, Working Alliance Short Version (Horvath and Greenberg, 1989; range = 12–60); Empathy = Therapist Empathy Scale (Decker et al., 2014; scale = 1–7); Warmth and Competence (Seewald and Rief, 2023a; scale = 1–5).

### Design and procedure

We measured outcome expectations four times as the primary dependent variable (see Figure 1): first (T0), at baseline, second (T1), after a standardized induction of negative outcome expectations via an audio presentation, third (T2), after a video showing an empathetic therapist (after first video), and fourth (T3), after the expectation-violation video aiming to improve the negative outcome expectations. The baseline assessment (T0) was excluded from the main analysis, because we wanted to assess how (inducted) negative outcome expectations can be violated. Therefore, we used a 3 (time: T1, T2, and T3) × 4 (group) online experimental design.

We used Sosci Survey (Leiner, 2019) for this online study. The experiment lasted around 25 min and the procedure can be seen in Figure 1. First, participants gave informed consent, exclusion criteria were checked, and the randomization was performed. After Baseline questionnaires (T0), an audio (2:08 min) was played that induced negative outcome expectations (“You have been told that therapy does not help most people in the long term.”) and participants imagined having experienced depressive symptoms (“Think of a time in your past when you were feeling very down and didn’t get as much done as usual”). We chose depressive symptoms because depression is the third leading cause of workplace absences in Germany (DAK-Gesundheit, 2023), affecting many people. Participants filled in questionnaires after the audio (T1) and were told to imagine they were attending an online therapy session for their symptoms. Before the video, a separate questionnaire page indicated whether the therapist would be human or an avatar. Participants watched the first of two videos (1:08 min) where a female therapist (*Human* or *Avatar*) expressed empathy for the depressive symptoms of the participant (“I can imagine that the last few weeks have not been easy for you and that you have felt lonely.”). Afterwards, participants filled in another questionnaire (T2), after which they were asked to imagine that the online therapy session was continuing and to watch the second video of the female therapist (*Human* or *Avatar*). Once again, the text on the page before the video mentioned whether the therapist would be human or an avatar. The second video (1:19 min) displayed an expectation violation of negative therapy expectations (“New studies show that the methods currently used to treat depression are quite effective and help most people”) by the therapist, again as a human therapist or as an avatar therapist. After the second video, participants filled in the last questionnaires (T3) and provided demographic data.

### Avatar therapist

The avatar was a photorealistic replica of a real female human therapist. The two videos showed the same person, either as a human therapist or as her photorealistic avatar. Unlike prior research, which often confounded visual stimulus characteristics with the disclosed identity of the agent (e.g., Dai and MacDorman, 2018; Seymour et al., 2020), our design keeps the appearance and voice of the therapist constant with only minimal behavioral peculiarities. Consequently, our manipulation successfully isolates the participants’ belief about the nature of the therapist from confounds in the stimulus material itself. The avatar videos were created using HeyGen software by entering text, which was then spoken by the avatar. While the photorealistic replica makes the avatar look human at first glance, upon closer inspection it becomes clear that the avatar displays repetitive facial expressions and hand movements. Furthermore, the facial expressions are not synchronized with the spoken text, but appear randomly.

Pretests involving two pairs of candidate therapist-avatars (*n* = 22 in each group) revealed significant main effects of both group (*F*(3, 36) = 4.67, *p* = 0.007) and actor (*F*(1, 36) = 4.41, *p* = 0.043) on empathy ratings. The former indicated that the four groups elicited different empathy ratings, while the latter showed that the first-person avatar elicited higher empathy ratings overall than the second. There was no significant interaction between actor and group, *F*(3, 36) = 0.86, *p* = 0.473, indicating that the two actors did not differ in how strongly the human-avatar manipulation affected perceived empathy. Based on their higher overall empathy ratings, the first therapist and their avatar were selected for the main experiment.

### Questionnaires

#### Main variables

*Outcome Expectations* were measured via the German Credibility Expectancy Questionnaire (CEQ; Devilly and Borkovec, 2000; Koch et al., 2016), comprising four 9-point Likert items (e.g., “At this point, how logical does the therapy offered to you seem?”) and two items ranging from 0 to 100% (transformed to a 9-point scale for analyses) that showed good internal consistency (*α* = 0.91). This variable was measured at baseline (T0), and before (T1) and after each of the two videos (T2, T3).

*Alliance* was measured via the German Working Alliance Inventory, Short Version (WAI-S, Horvath and Greenberg, 1989; Wilmers et al., 2008) with 12 items on a 5-point Likert scale (e.g., “My therapist and I work together to set therapy goals”) that showed good internal consistency (*α* = 0.93). This variable was measured after the first (T2) and second (T3) videos, respectively, since a therapeutic alliance can only be established following a therapist’s display.

#### Variables for manipulation check at T2 (after video 1)

*Warmth* and *Competence* were measured via 12 items from the stereotype content model (SCM; Fiske et al., 2002; adapted by Seewald and Rief, 2023a) on a 5-point Likert scale (e.g., “friendly,” “capable”) that showed good internal consistency for warmth (*α* = 0.94) and competence (*α* = 0.94).

*Empathy* was measured via a German translation of the Therapist Empathy Scale (TES; Decker et al., 2014) with nine items on a 7-point Likert scale (e.g., “To what extent were you able to perceive emotional understanding in the therapist in the video?”) that showed good internal consistency (*α* = 0.93).

#### Control variables

Depressive symptoms over the past 2 weeks were assessed using the German *Patient Health Questionnaire* (PHQ-9; Kroenke and Spitzer, 2002; Gräfe et al., 2004). The nine 4-point Likert items (e.g., “Little interest or pleasure in your activities”) showed good internal consistency (*α* = 0.86). We calculated a sum score (range: 0–27) with 10 as the clinical cut-off point.

*Technology Commitment* (Neyer et al., 2012) was measured by 12 items on a 5-point Likert scale (e.g., “I am very curious about new technical developments.”) that showed good internal consistency (*α* = 0.86).

*Credibility of the fictional scenario* (Mayer et al., 2024) was measured with a single item (“Was there at least one moment when you felt that the situation in this study could be real?”) that ranged from 1 to 101.

### Preregistered variables

*Attitudes Toward Seeking Professional Psychological Help* were measured by the German version (Coppens et al., 2013) of the ATSPPH (Fischer and Farina, 1995) with 10 items on a 4-point-Likert scale (e.g., “I would want psychological help if I was worried or distressed for a long time.”) that showed acceptable internal consistency (*α* = 0.82).

*Therapy Motivation* was measured by a single item (“How motivated are you to do psychotherapy to work on your problem?”) that ranged from 1 to 11 and was used before by Seewald and Rief (2023a).

### Statistical analyses

We used RStudio for all analyses (Version 4.4.2). We calculated Cronbach’s alpha for all main variables to check internal consistency and used linear models to test for group differences in the manipulation check variables warmth and empathy. Moreover, we used likelihood ratio tests to test nested linear models (with and without interaction effects of group and time) against each other (package lmrTest, function lrtest; Kuznetsova et al., 2017). A significant effect of the likelihood ratio test shows a better fit for the more complex model with the interaction effect and can be interpreted similarly to analyses of variance (ANOVAs). Due to randomization, we do not expect any baseline variable distributions that are disproportionate beyond chance. The normal distribution of the residuals of level 1 and 2 for the outcome expectation and alliance models was graphically satisfied. Due to the violation of homoscedasticity in level 1 residuals in the outcome expectation and alliance models, robust standard errors (CR2-estimator, clubSandwich, Pustejovsky and Tipton, 2018, 2023) were calculated to ensure the validity of the inference statistics. For the pairwise comparisons of each group with the reference group *Human_Human* at time point T3, we applied a Holm correction (Holm, 1979) to the CR2-robust contrast *p*-values within each outcome domain (three contrasts each for outcome expectations, alliance, and motivation; (linear_contrast, clubSandwich)). We used Bayes factors to indicate support for the null hypothesis (BF_01_) and for the alternative hypothesis (BF_10_ = 1/BF_01_) (BayesFactor package, Version 0.9.12–4.6; Morey and Rouder, 2023). For the fixed effects, the standard prior (a Zellner–Siow-inspired g-prior, *r* = 0.5; ‘medium’, Rouder et al., 2012) was retained. One convention is that Bayes Factors between 3 and 20 indicate positive evidence against the null hypothesis (Kass and Raftery, 1995).

We predicted a time × group interaction effect on outcome expectations and working alliance. Specifically, we expected the highest increase after the two videos in the *Human_Human* group, followed by the *Human_Avatar* group, the *Avatar_Human* group, and the *Avatar_Avatar* group (*Human_Human* > *Human_Avatar* > *Avatar_Human* > *Avatar_Avatar*).

## Results

### Manipulation checks

We predicted that the human empathy video significantly predicts warmth and empathy scores at T2 (after video 1). The linear model for warmth showed no significant differences between the human and avatar groups at T2 in warmth (*F*(1,482) = 0.79, *p* = 0.37, BF_10_ = 0.15). Nevertheless, the linear model for empathy showed the expected group differences between human and avatar groups (*F*(1,482) = 45.69, *p* < 0.001, BF_10_ = 185358269).

### Main analyses

#### Outcome expectations

The analysis included timepoints T1 (after audio), T2 (after video 1), and T3 (after video 2) for factor time. The pre-test (*N* = 22) showed a significant reduction of outcome expectations (*t*(21) = −5.82, *p* < 0.001) between the pre (T0) and post (T1) audio assessments which indicates a successful induction of negative outcome expectations.

In the main study, groups changed significantly in their CEQ scores from T1 (after audio) to T3 (after video 2) indicating an expectation violation of negative outcome expectations (*B* = 1.08, SE = 0.09, *t*(483) = 12.22, *p* < 0.001). The interaction between time and group significantly improved model fit, as indicated by a likelihood ratio test (*X*^2^(6) = 30.45, *p* < 0.001, *η_p_*^2^ = 0.03, BF_10_ = 179.29).

The linear mixed-effects model revealed significant interaction effects at T3 (after video 2) for both the *Human_Avatar* and *Avatar_Avatar* groups relative to the reference group *Human_Human* (see Figure 2). Specifically, the *Human_Avatar* group showed a significantly smaller increase in CEQ ratings from T1 (after audio) to T3 (after video 2) compared to the *Human_Human* group (*B* = −0.85, S*E* = 0.26, *t*(235) = −3.26, *p* = 0.004). Similarly, the *Avatar_Avatar* group showed a significantly smaller increase in CEQ ratings from T1 (after audio) to T3 (after video 2) relative to the *Human_Human* group (*B* = −0.58, SE = 0.24, *t*(238) = −2.38, *p* = 0.036).

**Figure 2:**
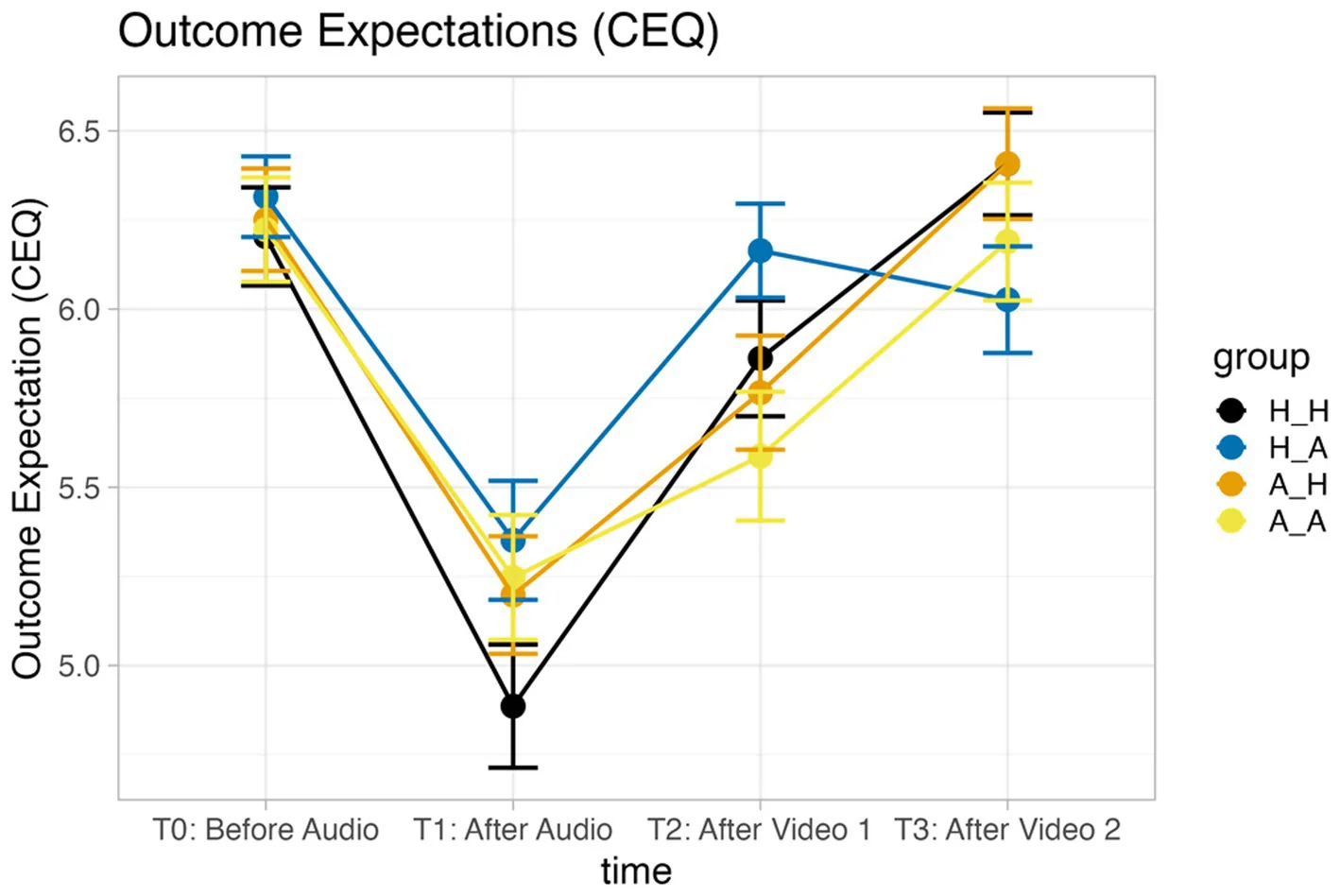
Mean Outcome Expectations (CEQ) per group. This figure displays the mean Outcome Expectation (CEQ, Credibility Expectancy Questionnaire, range = 1–9) per group for each timepoint. Abbreviations for the groups: H_H = *Human_Human*, H_A = *Human_Avatar*, A_H = *Avatar_Human*, A_A = *Avatar_Avatar*.

#### Working alliance

The interaction between time and group resulted in a significant improvement in model fit, as indicated by a likelihood ratio test (*X*^2^(3) = 53.54, *p* < 0.001 *η_p_*^2^ = 0.10, BF_10_ = 482,467,907). The linear mixed-effects model showed an interaction effect for the *Human_Avatar* group and the reference group (*Human_Human*) at T3 (after video 2) (see Figure 3). While the *Human_Human* group remained stable from T2 (after video 1) to T3 (after video 2), the *Human_Avatar* group showed a comparatively greater decline, resulting in significantly lower alliance scores at T3 (after video 2) (*B* = −4.41, SE = 0.91. *t*(235) = −4.86, *p* < 0.001). Moreover, there was an interaction effect for the *Avatar_Human* group and the reference group (*Human_Human*) at T3 (after video 2). While the *Human_Human* group remained stable in alliance ratings, the *Avatar_Human* group increased significantly more from T2 (after video 1) to T3 (after video 2) in their alliance scores (*B* = 1.97, SE = 0.80, *t*(237) = 2.47, *p* = 0.029).

**Figure 3:**
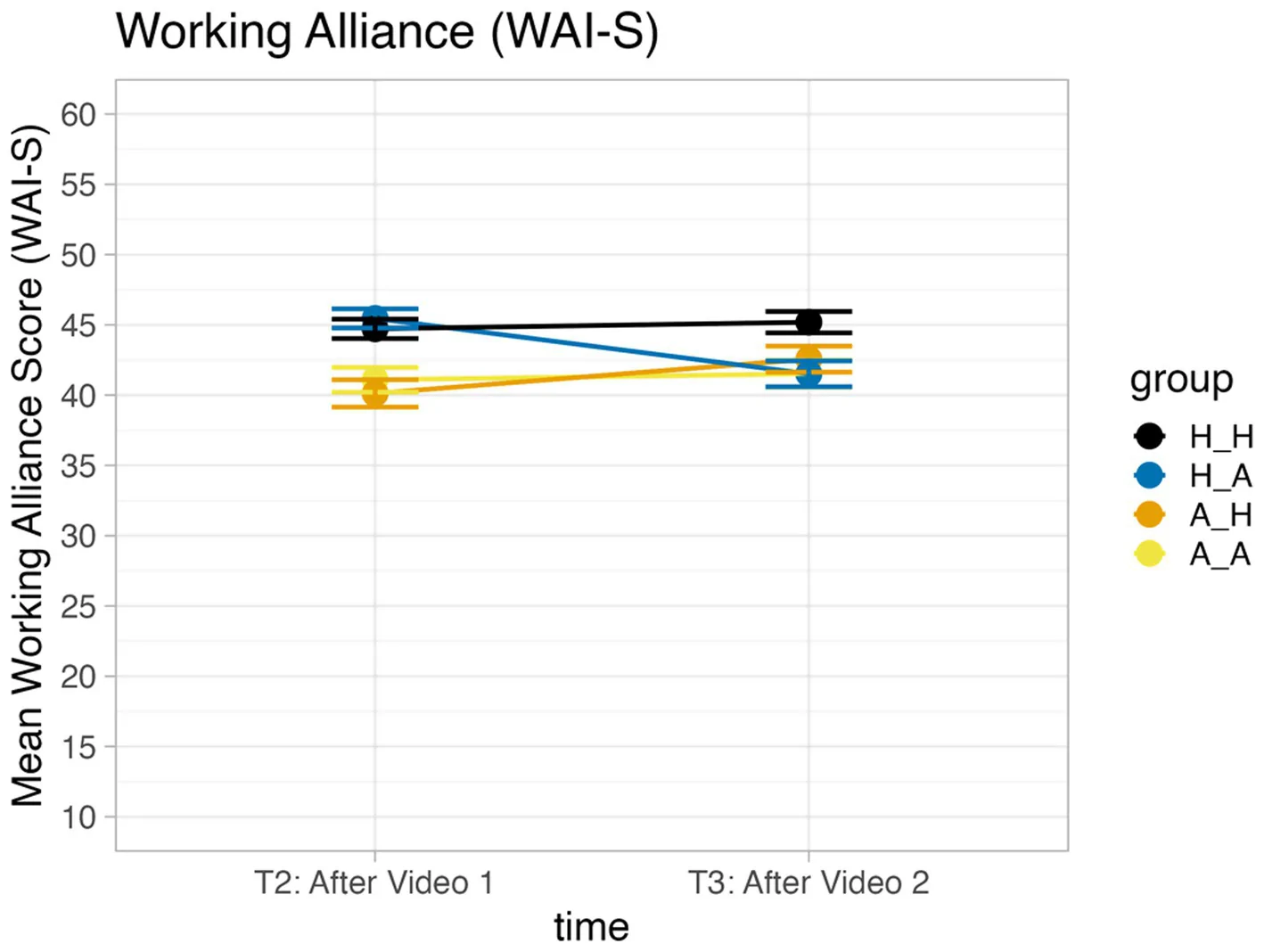
Mean Working Alliance (WAI-S) per group. This figure displays the mean Alliance (WAI-S, Working Alliance Questionnaire, Short Version, range = 12–60) per group for each timepoint. Abbreviations for the groups: H_H = *Human_Human*, H_A = *Human_Avatar*, A_H = *Avatar_Human*, A_A = *Avatar_Avatar*.

### Preregistered confirmatory analyses

#### Mediation analysis

For the mediation analyses, we used a structural equation model (SEM) (lavaan package, Rosseel, 2012) in R with maximum likelihood estimation and bootstrapping (*n* = 10,000). Indirect effects of all four groups are considered significant if the 95% confidence interval does not include zero. We compared a simple mediation model that used the same parameter for indirect effects across all four groups, to a complex model that allowed for a different parameter for the indirect effect for each group. A significant effect of the likelihood ratio test shows a better fit for the more complex mediation model. We predicted that the working alliance after video 1 partially mediates this relation, with the indirect effects being strongest in the *Human_Human group* compared to the other groups. This assumption could not be confirmed by the results.

When we compared the simple mediation with the more complex mediation with separate effects for each group, the more complex mediation model did not result in a significant improvement in model fit, as indicated by a likelihood ratio test (*X*^2^(9) = 13.18, *p* = 0.155) (AIC = 5081.491, BIC = 5160.950, RMSEA = 0.06). Therefore, we used the simple mediation model without separate effects for the four groups. The analysis showed a significant indirect effect from *CEQ* after audio (T1) to *CEQ* after video *2* (T3) through the mediator alliance after video 1 (T2) (*B* = 0.09, *p* < 0.001, 95%-CI [0.05, 0.14]). CEQ after the audio (T1) significantly predicted alliance after video 1 (T2) (*a-path*; *b* = 0.93, *p* < 0.001, 95%-CI [0.48, 1.36]). Additionally, alliance after video 1 (T2) significantly predicted CEQ after video 2 (T3) (*b-path*; *b* = 0.10, *p* < 0.001, 95%-CI [0.09, 0.12]). The direct effect of CEQ after the audio (T1) to CEQ after video 2 (T3) was significant as well (*c′-path*; *b* = 0.28, *p* < 0.001, 95%-CI [0.21, 0.36]), which indicated a partial mediation. The change in outcome expectations before the videos (T1) to after the second video (T3) was mediated by alliance scores, independently of group. Since the indirect effect did not differ between the groups, this mediation result should be understood as reflecting a chronological, cross-group relationship between earlier outcome expectations, therapeutic alliance, and later outcome expectations, rather than as an explanation for why the different sequences of human and avatar videos produced different results.

#### Other preregistered confirmatory analyses

The time × group interaction model of Attitudes Towards Seeking Professional Psychological Help did not result in a significant improvement in model fit compared to the additive model. The time × group interaction model of therapy motivation significantly improved model fit (*X*^2^(3) = 28.21, *p* < 0.001, *η_p_*^2^ = 0.06, BF_10_ = 3237.96). The linear mixed-effects model revealed that at T3 (after video 2), all three groups (*Human_Avatar*, *Avatar_Human*, *Avatar_Avatar*) showed a smaller increase in motivation from T1 (after audio) to T3 (after video 2) compared to the reference group (*Human_Human*), resulting in significantly lower motivation scores at T3 (after video 2). Technology commitment and age did not show significant effects as covariates in the models of outcome expectations and alliance. Covariate analyses showed that high scores on credibility of the fictional scenario were associated with higher outcome expectations and higher alliance ratings. No previous treatment for depression was associated with higher alliance ratings. Competence showed an interaction effect in the *Human_Avatar* group for outcome expectation and alliance. This indicates that the positive association between perceived competence and outcome expectations (respectively, alliance) was significantly weaker in the *Human_Avatar* group compared to the *Human_Human* reference group. All preregistered analyses can be found in the Supplemental material B.

#### Exploratory text analysis without preregistration

We also examined the optional open comment field at the end of the study more closely (“Do you have any comments about the study that you would like to share with us?”). A total of 66 participants (13.64%) provided an answer in the free-text field. Twenty-seven (40.90% of *N* = 66) of the replies were independent of the study topic (e.g., “No”; “None”; “Good luck”; “Very interesting study”). Sixteen participants (24.24% of *N* = 66) commented on the therapists shown in the video, expressing some confusion as to whether they were avatars or human therapists. (e.g., “[…] Both videos showed human therapists, but the text above first said ‘human’ and then “avatar” for the second one.”; “In the first video, I was told it was an avatar, but I’ve watched two videos featuring the same (human) therapist. I’m a little confused.”; “They said the second video showed a human therapist—was that really the case? To me, the therapist still seemed a bit like an AI […]”; “I really had a hard time figuring out whether the therapist was an avatar or not. If it was an avatar, it was very well done.”).

## Discussion

This online experiment examined the effects of human versus avatar therapist videos on outcome expectations and therapeutic alliance. After induction of negative outcome expectations in all four groups, an initial empathic video was followed by a video challenging negative outcome expectations by an actor being either human or a photorealistic avatar-therapist. In line with our hypotheses (*Human_Human* > *Human_Avatar* > *Avatar_Human* > *Avatar_Avatar)*, the expectation violation and therapeutic alliance were highest in the *Human_Human* group, where empathy and expectation violation were both delivered by the human therapist. Moreover, outcome expectations and alliance ratings increased in the *Avatar_Human* and *Avatar_Avatar* groups after video two. Contrary to our hypotheses, outcome expectations and therapeutic alliance in the *Human_Avatar* group declined rather than continued to rise, following exposure to the expectation violation video of the avatar-therapist. Due to the drop in the *Human_Avatar* group, the empirical ranking deviated from the hypothesized linear order, namely *Human_Human* > *Avatar_Human* > *Avatar_Avatar* > *Human_Avatar*, with outcome expectations being significantly lower in the *Human_Avatar* and *Avatar_Avatar* groups compared to the *Human_Human* reference group.

The unexpected decline in the *Human_Avatar* group after video two (expectation violation video by an avatar) could be interpreted as a contrast effect. The pattern does not support our initial assumption that a prior alliance with a human-therapist would potentiate the effect of an expectation violation through an avatar-therapist, but rather suggests the opposite. Our findings indicate that the impact of photorealistic avatars in psychotherapeutic contexts is dependent on the sequence of presenting human and avatar-based information: whereas transitioning from human to avatar (*Human_Avatar*) yielded the lowest alliance and outcome expectation scores, beginning with the avatar and transitioning to a human (*Avatar_Human*) produced rising outcome expectations and alliance. Of note, such a negative sequence effect was not observed for the *Avatar_Avatar* group. This asymmetry helps determine the two accounts of the *Human_Avatar* decline. A pure content-modality account—whereby avatars are less effective at delivering expectation violation content—predicts weak or negative effects whenever an avatar delivers video 2. In the *Avatar_Avatar* group, outcome expectations continued to rise from after the first to after the second video, though this increase was smaller than in the *Human_Human* group. The corresponding increase of alliance in *Avatar_Avatar* was not significant. We interpret this pattern as most consistent with a modest, general content-modality-related attenuation present whenever an avatar delivers video 2, evident in the smaller increases in both avatar-second-video groups relative to *Human_Human*, and an additional sequence-specific contrast effect that produces an outright reversal only when the avatar follows a human therapist (*Human_Avatar*).

One possible mechanism for the violation of expectations is the therapeutic alliance and, possibly in particular, the empathy that the participants perceived in the various videos. The manipulation check indicated that empathy was greater for the human empathy videos than for the avatar empathy videos. Even though participants reported significantly lower alliance scores after watching the avatar videos, the scores of the *Avatar_Avatar* group—at over 40 out of a possible 60 points—were not low per se. In contrast, higher levels of empathy in interactions with non-human entities were observed in other studies: Howcroft et al. (2025) found in their meta-analysis (*N* = 15) that AI-chatbots often exceed human healthcare professionals in text-based empathy assessments (*N* = 13). However, these findings were largely based on text-only interactions, single-item measures, and broad physical health conditions (Howcroft et al., 2025). This highlights the unique difficulty of conveying nonverbal empathy (e.g., nodding) in digital mental health contexts.

Although we expect higher alliance values for avatars compared to other digital entities in the current study, the avatar’s unsynchronized facial expressions and gestures may have diminished nonverbal signals of empathy. Switching from a human therapist to an avatar was accompanied by a reduction in non-verbal expressiveness, as well as a lack of synchronization, such as repetitive movements and facial expressions that were out of sync with speech. However, the study design does not allow for a distinction to be made between the effect of the change in the perceived ‘nature’ of the therapist (i.e., human versus avatar) and the effect of this reduced non-verbal quality. The observed decline in expectations and therapeutic alliance may therefore be primarily attributable to limited non-verbal communication rather than to the avatar category itself. In order to distinguish avatar-specific quality characteristics (e.g., synchrony and range of motion) from the human–avatar distinction, it would have been necessary to systematically manipulate these non-verbal characteristics.

Our alliance findings are for the most part consistent with the findings of other studies on blended formats. Although the study was designed differently from a methodological perspective, participants in an experimental vignette study showed highest trust, satisfaction, and information disclosure to a human doctor, followed by a human doctor supported by an AI system, followed by an AI system (i.e., chatbots) (Riedl et al., 2024). Their results additionally showed that for general medical contexts (orthopedics, cardiology, and dermatology), participants showed higher trust ratings for their interaction partners, whereas in the psychiatry condition, participants showed the lowest trust ratings (Riedl et al., 2024). This indicates that the therapeutic alliance is of heightened importance in psychiatric settings. Our study may provide additional sequential information for the psychotherapy context, namely, that contrast effects could occur in a blended format with different sequences of delivery (human vs. avatar), particularly after the formation of an initial *human* alliance.

The temporal pattern observed in our study—a disruption of alliance that emerges only at the second exposure in the Human_Avatar group—is not the only pattern reported for the presence-alliance relationship in avatar-based interventions. In a study of real-time, synchronized avatar counseling across a full treatment course, Im et al. (2024) found that therapeutic presence was most strongly linked to alliance in the earliest sessions. This effect faded by the final session, which is the opposite of our observed pattern. We make this comparison loosely, rather than viewing it as a contradiction, since the designs differ in features that could act as boundary conditions: single, brief, unsynchronized exposures versus a multi-session trajectory involving real-time, synchronized avatars and actual clients rather than analog participants. Future research should systematically vary these features—exposure count, avatar fidelity, and sample type—rather than treating divergent temporal patterns as competing accounts of the same phenomenon.

### Practical implications

In terms of clinical practice, the study could indicate recommendations for blended care models. The direct transition from a human-led session to an avatar-supported intervention may warrant caution, as this could jeopardize the therapeutic alliance. Conversely, avatars could be used as a low-threshold entry point for expectation violation of negative outcome expectations before starting the actual therapy, where a human therapeutic relationship can be established. Lungu et al. (2020) demonstrated in their blended-care CBT program a reduction of depressive symptoms (*d* = 1.59) after an average of 5.2 sessions, which is significantly shorter than a regular CBT program of 12–16 sessions. Their digital tools included lessons and exercises, and therapist sessions were conducted via video (Lungu et al., 2020). Hence, using avatars for, e.g., psychoeducation appears to be a next step forward for future studies. Crucially, the role of the avatar must be clearly communicated. Patients should be aware when they are interacting with an avatar, in order to avoid disappointment or mistrust.

Not all components of therapy should be outsourced to digital interventions. Van der Vaart et al. (2014) used a Delphi method, where depression experts (*N* = 21 therapists and patients) responded that practical therapy components such as diaries and psycho-education would be suitable for online modules, whereas process-related components such as evaluation and discussing thoughts and feelings should still be delivered via face-to-face therapy (Van der Vaart et al., 2014). This content-based distinction maps directly onto sequence design: if digital or avatar-led modules handle psychoeducation early, the human therapist can focus on relational work in later sessions. This shows that digital applications can be added to face-to-face therapy but should not substitute for psychotherapy entirely.

Crucially, acceptance rates of digital interventions vary by user group: while community samples readily accept avatars and chatbots, patients strongly preferred human-led teletherapy (human via screen) (Békés and Aafjes-van Doorn, 2026). As avatars reduce communication barriers (Rehm et al., 2016), they could be used to capitalize on community acceptance during the initial or low-threshold stages of a sequential design. In that way, users could be engaged by avatars before transitioning to a preferred human therapist. Nevertheless, digital interventions in general and specific avatars currently share a critical limitation: a persistent lack of deeply personalized, empathetic, and emotionally effective interactions (Anisha et al., 2024; Malouin-Lachance et al., 2025).

### Limitations

One important limitation in our study concerns the validity of the manipulation (human vs. avatar). Qualitative feedback from 16 participants showed that some participants were unsure whether they were watching a human or an avatar in the videos, despite corresponding pre-information. Some reported seeing two human therapists, despite acknowledging that they were told they would see an avatar. This raises the question of whether the observed effects are attributable to the avatar’s nature, or to participants’ failure to differentiate within the *uncanny valley* (i.e., Seymour et al., 2021) in a way that they did not recognize the avatars as non-human. If the avatar was perceived as *almost human* yet *somehow wrong* without being explicitly recognized as an avatar, then the negative effects may stem more from subtle confusion than from a conscious rejection of the avatar. Alternatively, our study possibly has crossed the *uncanny valley* where the photorealistic avatar was perceived as fully human. We conducted a sensitivity analysis in which the models for the outcome expectations and the therapeutic alliance were recalculated. Participants were excluded based on the qualitative item (*N* = 16), and the effects and their direction remained consistent.

The therapeutic scenario in this study was highly simplified (videos, no real interaction, no long-term relationship). In real psychotherapy, a therapeutic alliance is built over time and across many interactions. A switch from a human to an avatar in a 2-min video sequence is an abrupt transition. It is questionable whether these results are transferable to real, long-term blended care scenarios, where the transition might be smoother, or the avatar has a clearly defined role (e.g., coach) rather than replacing the therapist. Relatedly, to ensure greater external validity and increase generalizability, it would be important to study additional human-avatar pairs. The asynchrony between the avatar and human may differ between pairs.

After the first video, there was no significant difference in warmth ratings between the human and the avatar videos. Only empathy was higher for the human video after the first video. Empathy and warmth are usually closely linked, responding together to non-verbal manipulation (e.g., Maaß and Weck, 2026; Kraft-Todd et al., 2017). Therefore, our finding of a difference is surprising. This difference may reflect the different depths at which empathy and warmth are measured. Items measuring empathy, such as “concern” and “attunement” may reflect a deeper level of warm behavior than the more superficial behavioral descriptions of warmth, such as “friendly,” “good-natured.” Future studies could test this by examining whether the differences between avatars and humans are primarily evident in empathy items that require a deeper level of understanding, or rather in items that are more behaviorally oriented.

A further limitation is that we studied a relatively well-educated sample with a low burden of depressive symptoms. This limits our ability to draw conclusions about the potential clinical implications of these findings.

The order of the videos was fixed: the first always contained empathetic validation and the second contained expectation violating information. However, whether a video was presented by the human therapist or her avatar was randomized independently of this. While the sequence effect cannot be attributed to confounding factors relating to the type of therapist, it remains unclear whether it reflects the position of the video (first vs. second) or the content (empathy vs. unmet expectations), since the two factors are confounded. Future studies should experimentally vary the order of the content to distinguish between position and content effects.

When interpreting the changes of outcome expectations following the second video, one must consider that outcome expectation scores after the first video (T2) had largely returned to baseline levels (T0). As negative expectations had already been substantially reduced by this point, there was limited scope for further improvement after video 2 (T3). Therefore, minimal or no changes following the second video should not immediately be interpreted as an absence of effect, but may in part be attributable to this limited scope for change (floor effect).

## Conclusion

Photorealistic avatars can violate negative outcome expectations in healthy participants, but this may be most effective when avatars are used prior to videos of human expectation violations rather than when humans are used prior to avatars. Though, the design cannot fully disentangle whether this reflects besides the sequence of avatar exposure, a general tendency for avatars to be less effective at delivering expectation violations. In terms of outcome expectations and alliance ratings, human videos outperformed photorealistic avatar videos in this study; however, because the avatar’s facial expressions and movements were repetitive and not fully synchronized with speech, this difference cannot be unambiguously attributed to the human-avatar distinction itself rather than to this specific avatar’s non-verbal quality. Conclusions about blended-care practices should be drawn cautiously, and broad recommendations to avoid transitions from human to avatar therapists are not yet warranted. Our findings highlight the importance of transparency regarding the new possibilities offered by photorealistic avatars when designing blended-care concepts. Clearly labeling content of avatars versus that of human therapists could help to mitigate the uncanny valley effect. In addition, photorealistic avatars could strengthen the therapeutic bond through their human-likeness (anthropomorphism). Future research should systematically vary avatar-specific quality characteristics (e.g., synchrony) independently of the human-avatar distinction, and examine whether these effects replicate with clinical samples and in ecologically valid therapeutic contexts.

## Data Availability

The experiment’s design, hypotheses, and analysis plans were preregistered at the Open Science Framework OSF: https://doi.org/10.17605/OSF.IO/B7W5A. The R-code for the analyses can be found at https://osf.io/agu3k/files/pdct3.
