# Supplementary material for: Photorealistic avatars can help to improve treatment outcome expectations
Source: Front Psychol. 2026 Oct 21;17:1960478. doi: 10.3389/fpsyg.2026.1960478 (PMC13732318; doi:10.3389/fpsyg.2026.1960478)
Supplement: Supplementary file 1 [file Supplementary_file_1.docx]

**Supplement**

**A**

**Figure 1**

*CONSORT Diagram of Participant Flow*

**B**

**Pre-registered Confirmatory Analyses**

**Attitudes Towards Seeking Professional Psychological Help**

We predicted that the *Human_Human* group will have the highest attitudes towards seeking professional psychological help after the two videos followed by the *Human_Avatar* group, the *Avatar_Human* group, and the *Avatar_Avatar* group. Therefore, we predict an interaction effect of time x group (*Human_Human* > *Human_Avatar* > *Avatar_Human* > *Avatar_Avatar*). The interaction between time and group resulted in no significant improvement in model fit, as indicated by a likelihood ratio test (*X*^2^(3) = 6.39, *p* = .094 η_p_ ^2^ = .01, BF_10_ = 0.14).

**Therapy Motivation**

We predicted that the *Human_Human* group will have the highest therapy motivation after the two videos followed by the *Human_Avatar* group, the *Avatar_Human* group, and the *Avatar_Avatar* group. Therefore, we predict an interaction effect of time x group (*Human_Human* > *Human_Avatar* > *Avatar_Human* > *Avatar_Avatar*). The interaction between time and group resulted in a significant improvement in model fit, as indicated by a likelihood ratio test (*X*^2^(3) = 28.21, *p* < .001, η_p_ ^2^ = .06, BF_10_ = 3237.96).

The linear mixed-effects model showed an interaction effect for the *Human_Avatar* group and the reference group (*Human_Human*) at T3. While the *Human_Human* group showed a greater incline from T1 to T3, the *Human_Avatar* group showed a comparatively smaller incline, resulting in significantly lower motivation scores at T3 (B = -1.92, S*E* = 0.37, *t*(235) = -5.14, *p* < .001) A similar pattern of interaction appeared for the *Avatar_Human* and *Avatar_Avatar* group compared to the reference group (*Human_Human*) at T3. While the *Human_Human* showed a greater incline in motivation ratings, the *Avatar_Human* and *Avatar_Avatar* group showed a comparatively smaller incline from T1 to T3 in their motivation scores (B = -1.26, S*E* = 0.35, *t*(237) = -3.66, *p* < .001 and B = -1.11, S*E* = 0.34, *t*(238) = -3.28, *p* = .001 respectively).

**Figure 2**

*Mean Therapy Motivation per group*

*Note*. Figure 2 displays the mean Therapy Motivation (Seewald and Rief, 2023, range = 1–11) per group for each timepoint. Abbreviations for the groups: H_H = *Human_Human*, H_A = *Human_Avatar*, A_H = *Avatar_Human*, A_A = *Avatar_Avatar.*

**Preregistered Exploratory Analyses**

**Technology Commitment and Age**

We predicted that older individuals and those with lower technology commitment will show smaller effects in the avatar-groups for outcome expectations and alliance. To test this hypothesis, we extended the two multilevel model of outcome expectations and alliance by adding interaction terms between group and each moderator (age and technology commitment, both z-standardized). None of the interaction effects for outcome expectations with either age (b = -0.12, SE = 0.20, *t* (472) = -0.58, *p* = 0.561) or technology commitment (b = 0.28, SE = 0.20, *t* (472) = 1.39, *p* = 0.165) reached statistical significance for the avatar-groups indicating no support for our hypothesis. Similar, nonsignificant interaction effects were found for alliance and age (b = -0.84, SE = 1.20, *t* (472) = -0.70, *p* = 0.485) and technology commitment (b = 1.66, SE = 1.18, *t* (472) = 1.40, *p* = 0.162).

**Table 1**

Neuroticism, ATSPPH and Motivation at Each Point of Time for the Four Groups

|  | Human Human | Human Avatar | Avatar Human | Avatar Avatar | | |
| --- | --- | --- | --- | --- | --- | --- |
|  | *n* = 117 | *n* = 120 | *n* = 123 | *n* = 124 | | |
| **ATSPPH** *M* (*SD)* |  |  |  |  | | |
| T1 | 18.09 (5.71) | 18.63 (4.77) | 19.20 (4.68) | | 18.55 (5.78) | |
| T3 | 20.69 (5.20) | 19.90 (4.11) | 20.98 (4.50) | | 20.16 (5.80) | |
| **Motivation** *M* (*SD)* |  |  |  | |  | |
| T1 | 4.17 (2.80) | 5.31 (2.95) | 5.15 (2.80) | | | 4.89 (3.10) |
| T3 | 6.73 (2.26) | 5.94 (2.50) | 6.44 (2.56) | | | 6.33 (2.70) |
| **Neuroticism** | 3.05 (0.96) | 2.94 (0.97) | 2.87 (1.03) | | | 2.94 (1.08) |

*Note.* ATSPPH = Attitudes Toward Seeking Professional Psychological Help-Short Form (Fischer & Farina, 1995; range = 0–30); Motivation (Seewald & Rief, 2023; range = 1–11); Neuroticism (BFI-10, Rammstedt & John, 2007; scale = 1–5).

**Covariate Analysis**

Neuroticism (Rammstedt & John, 2007) was measured by two items of the Big Five Inventory (BFI-10) on a 5-point Likert scale (e.g., “I see myself as someone who gets nervous easily”) that showed sufficient internal consistency (α = .71).
We preregistered to investigate, whether demographic variables (gender and education), depression, previous treatment experience, the credibility of the fictional scenario, and personality variables have an impact on the models of outcome expectations and alliance. None of the variables had a significant effect on *outcome expectations*, except credibility of the fictional scenario (B = 0.48, SE = 0.07, *t* (467) = 7.26, *p* < 0.001). This means that participants who found the scenario more credible, generally had higher outcome expectations. None of the variables had a significant effect on *alliance*, except credibility of the fictional scenario (B = 3.56, SE = 0.38, *t*(467) = 9.39, *p* < 0.001) and no previous depression treatment (B = 2.06, SE = 1.01, *t* (467) = 2.04, *p* = 0.041). This means that participants who found the scenario more credible, showed higher alliance, and those who had not previously received therapy for depression showed higher alliance in general.

Exploratorily, we conducted a group comparison for the credibility of the fictional scenario item and found a significant effect for the *Avatar_Avatar* group compared to the *Human_Human* reference group (B = -8.48, SE = 4.10, *t*(480) = -2.07, *p* = 0.039). This means that participants of the *Avatar_Avatar* group rated the experiment as less credible.

**Exploratory Analysis without Preregistration**

**Perceived Competence in Outcome Expectation and Alliance models**

We examined the effects of perceived competence of the (avatar-) therapist on our models of outcome expectations and alliance. The interaction between group and competence on *outcome expectations* was significant for the *Human_Avatar* group (B = -0.38, SE = 0.19, *t* (476) = -1.98, *p* = 0.048). This indicates that the positive association between perceived competence and outcome expectations was significantly weaker in the *Human_Avatar* group compared to the *Human_Human* reference group.

The interaction between group and competence on *alliance* was significant for the *Human_Avatar* group (B = -2.13, SE = 0.89, *t* (476) = -2.38, *p* = 0.018). This indicates that the positive association between perceived competence and alliance was significantly weaker in the *Human_Avatar* group compared to the *Human_Human* reference group.

**C**

*Audio Induction of depressive symptoms and negative outcome expectations*

Think of a time in your past when you were feeling very down and weren’t getting as much done as usual. Imagine that this state—in which you’re not functioning at your best—has now been going on for quite some time. You’re having a hard time concentrating on everyday tasks, and you’re wondering how you’re supposed to get everything done. On top of that, you’re not sleeping as well, (see Luftey et al., 2009) which is further affecting your performance. You’re doubting yourself, and the things that usually make you feel better aren’t helping right now (see Klineberg et al., 2011). You’re afraid things will just keep getting worse. You’re withdrawing and reaching out less to the people who are actually important to you. You feel lonely, but you just can’t bring yourself to reach out more. You decide you want to do something about it and search the internet for an explanation for how you’re feeling. You realize that what you’re experiencing are symptoms of depression. You decide to seek help and start chatting in a forum with other people who suffer from depression. You had hoped to finally find helpful tips and strategies there so you wouldn’t be at the mercy of your unpleasant feelings anymore, but that hope was not fulfilled. You didn’t get the help you needed; instead, you learned that therapy hadn’t helped other people and that depression often returns even after therapy. You found a study claiming that therapy didn’t help 70% of people with depression (*effectiveness*, Seewald & Rief, 2023). Recently, you also heard from a close friend that therapy didn’t help her. She’s been struggling helplessly with her depression for years now (*treatment success*, see Seewald & Rief, 2023). Your friend tells you that depression is quite persistent and difficult to overcome (*treatment rationale and reference level*, Seewald & Rief, 2023).

*Empathy video script*

You’ve mentioned that you feel hopeless, have trouble sleeping and concentrating, and are doubting yourself more often. I can imagine that the past few weeks haven’t been easy for you and that you’ve felt lonely. It must be frustrating when your concentration isn’t as good as it used to be and everything takes more effort than usual. That makes it all the more remarkable to me that you’ve decided to be here today and give psychotherapy another chance! It’s very important to me that we have a trusting atmosphere where you feel comfortable addressing issues early on. If you give me honest feedback about how you’re doing in therapy, we can work well together. I am confident that we can help you if we tackle your problems together. We will first look at exactly where we can start with therapy so that you feel better. To do this, I would like to ask a few more questions about what you have shared so far to make sure I have understood you correctly.

*Expectation violation video*

Psychotherapists used to believe that depression was a permanent condition, but today it is generally accepted that therapy can help manage depression. There is a wide range of research on depression. Recent studies show that the methods currently used to treat depression are quite effective and help most people (*effectiveness*, Seewald & Rief, 2023). Therapy can therefore be helpful when done correctly. Approximately 5.3 million adults in Germany struggled with a depressive disorder last year, which is about 8%. Depression is therefore a fairly common condition. Psychotherapy has helped many people who were in a situation similar to yours (*reference level*, Seewald & Rief, 2023. Former patients report that they felt better after therapy and learned how to cope with their problems (*treatment success*, Seewald & Rief, 2023). In psychotherapy, the therapist combines various methods and selects the ones that best suit the individual’s specific issues (*treatment rationale*, Seewald & Rief, 2023). Depending on the symptoms present, the therapy goals are established at the beginning, and then a corresponding treatment plan is created.

**References**

Fischer, E. H., & Farina, A. (1995). Attitudes toward seeking professional psychological help: A shortened form and considerations for research. *Journal of College Student Development*, 36 (*4*), 368–373. <https://doi.org/10.1037/t05375-000>

Klineberg, E., Biddle, L., Donovan, J., & Gunnell, D. (2011). Symptom recognition and help seeking for depression in young adults: A vignette study. *Social Psychiatry and Psychiatric Epidemiology*, *46*(6), 495–505. <https://doi.org/10.1007/s00127-010-0214-2>

Lutfey, K. E., Link, C. L., Grant, R. W., Marceau, L. D., & McKinlay, J. B. (2009). Is certainty more important than diagnosis for understanding race and gender disparities?: An experiment using coronary heart disease and depression case vignettes. *Health Policy*, *89*(3), 279–287. <https://doi.org/10.1016/j.healthpol.2008.06.007>

Rammstedt, B., & John, O. P. (2007). Measuring personality in one minute or less: A 10-item short version of the Big Five Inventory in English and German. *Journal of Research in Personality*, *41*(1), 203–212. <https://doi.org/10.1016/j.jrp.2006.02.001>

Seewald, A., & Rief, W. (2023). How to Change Negative Outcome Expectations in Psychotherapy? The Role of the Therapist’s Warmth and Competence. *Clinical Psychological Science*, *11*(1), Article 1. <https://doi.org/10.1177/21677026221094331>
